# A case report of reversible generalized seizures in a patient with Waardenburg syndrome associated with a novel nonsense mutation in the penultimate exon of *SOX10*

**DOI:** 10.1186/s12887-018-1139-2

**Published:** 2018-05-23

**Authors:** Noriomi Suzuki, Hideki Mutai, Fuyuki Miya, Tatsuhiko Tsunoda, Hiroshi Terashima, Noriko Morimoto, Tatsuo Matsunaga

**Affiliations:** 1 Department of Otolaryngology, National Center for Child Health and Development, Tokyo, Japan; 2 Division of Hearing and Balance Research, National Institute of Sensory Organs/ Medical Genetics Center, National Hospital Organization Tokyo Medical Center, 2-5-1 Higashigaoka, Meguro, Tokyo, 152-8902 Japan; 3 Department of Medical Science Mathematics, Medical Research Institute, Tokyo Medical and Dental University, Tokyo, Japan; 4 Laboratory for Medical Science Mathematics, RIKEN Center for Integrative Medical Sciences, Yokohama, Japan; 5 Devision of Neurology, National Center for Child Health and Development, Tokyo, Japan

**Keywords:** SOX10, Waardenburg syndrome, Nonsense mutation, Delayed myelination, Seizure attack

## Abstract

**Background:**

Waardenburg syndrome type 1 (WS1) can be distinguished from Waardenburg syndrome type 2 (WS2) by the presence of dystopia canthorum. About 96% of WS1 are due to *PAX3* mutations, and *SOX10* mutations have been reported in 15% of WS2.

**Case presentation:**

This report describes a patient with WS1 who harbored a novel *SOX10* nonsense mutation (c.652G > T, p.G218*) in exon 3 which is the penultimate exon. The patient had mild prodromal neurological symptoms that were followed by severe attacks of generalized seizures associated with delayed myelination of the brain. The immature myelination recovered later and the neurological symptoms could be improved. This is the first truncating mutation in exon 3 of *SOX10* that is associated with neurological symptoms in Waardenburg syndrome. Previous studies reported that the neurological symptoms that associate with WS are congenital and irreversible. These findings suggest that the reversible neurological phenotype may be associated with the nonsense mutation in exon 3 of *SOX10*.

**Conclusions:**

When patients of WS show mild prodromal neurological symptoms, the clinician should be aware of the possibility that severe attacks of generalized seizures may follow, which may be associated with the truncating mutation in exon 3 of *SOX10*.

**Electronic supplementary material:**

The online version of this article (10.1186/s12887-018-1139-2) contains supplementary material, which is available to authorized users.

## Background

Waardenburg syndrome (WS) is a hereditary disease characterized by sensorineural hearing loss and pigmentation abnormalities. WS has been classified into four subtypes on the basis of the clinical symptoms, namely, WS1 to WS4 [1]. Mutations in six genes (*PAX3*, *MITF*, *EDN3*, *EDNRB*, *SOX10*, and *SNAI2*) have been reported as the cause of WS [1].

Mutations in *SOX10* were first reported to associate with WS4 and then later with WS2. WS4 can be accompanied by neurological symptoms, in which case it is called PCWH (Peripheral demyelinating neuropathy, Central dysmyelinating leukodystrophy, Waardenburg syndrome, Hirschsprung disease, OMIM: #609136) [2]. While WS2 can also be accompanied by neurological symptoms, only six cases have been reported to date [3–5]. Those reports suggest that the neurological symptoms that associate with WS are congenital and irreversible.

In this report, we describe the case of a patient with WS1 who harbored a de novo *SOX10* heterozygous nonsense mutation. The patient had mild prodromal neurological symptoms that were followed by an attack of generalized seizures that associated with delayed myelination of the brain which recovered later.

## Case presentation

**Fig. 1:**
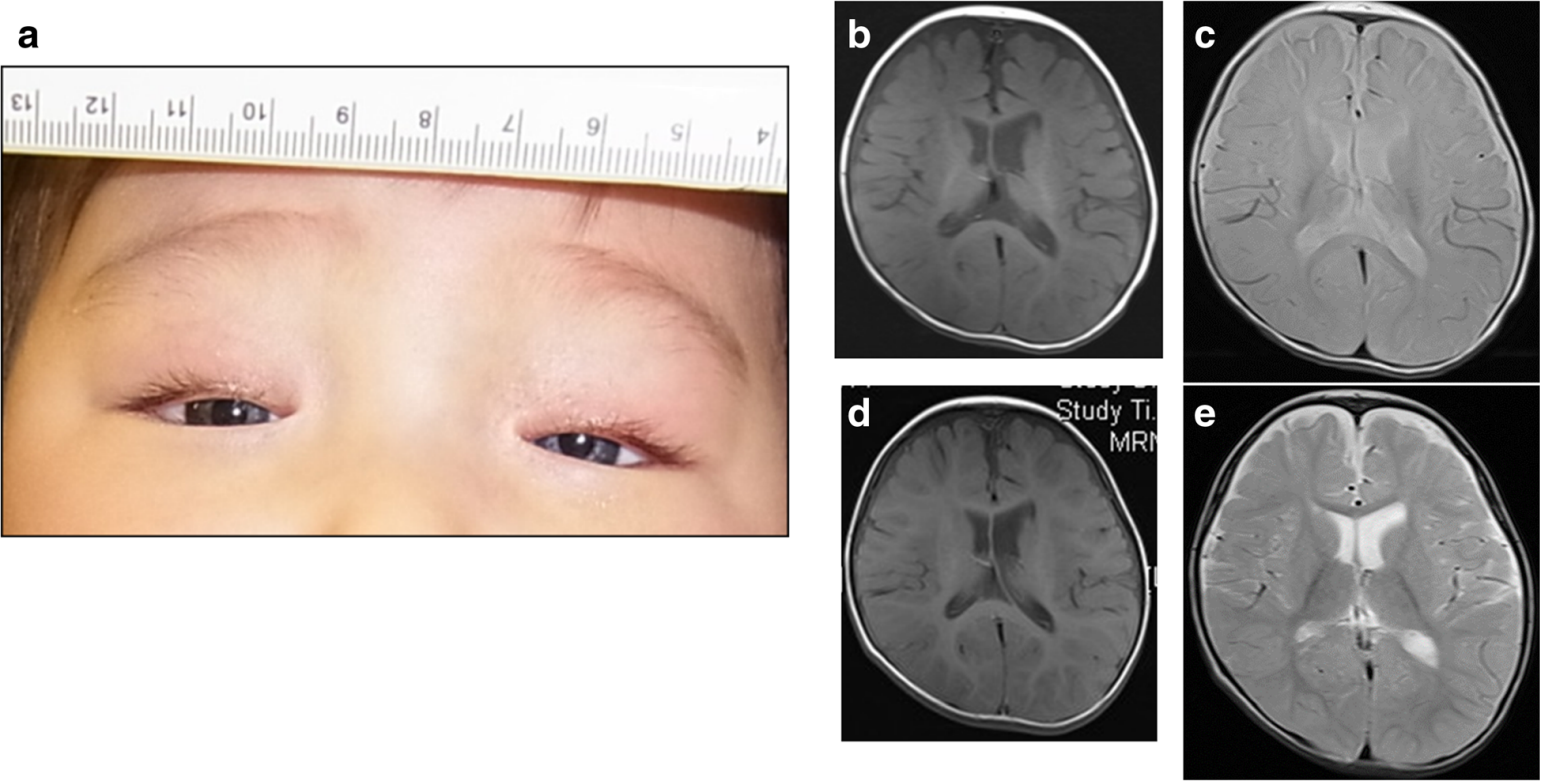
Clinical features of the patient. **a** The face at 5 months of age. The patient had blue irides, blepharoptosis, and dystopia canthorum (W-index: 2.24). **b**-**e** Brain MRI findings at 8 and 18 months of age. At 8 months of age, namely, 3 months after the proband had a 4-min-long systemic clonic seizure, T1- **b** and T2-weighted images **c** demonstrated delayed myelination of the frontal lobe. In normal development, myelination of frontal lobe is observed at 3–4 months of age on T1-weighted imaging, and at 7–8 months of age on T2-weighted imaging. Follow-up MRI performed at 18 months of age demonstrated almost normal myelination on T1-weighted imaging **d** and T2-weighted imaging **e**

A 5-month-old boy was referred to our hospital with the chief complaint of congenital deafness. No abnormalities were observed during his perinatal course. However, at presentation, his motor development was delayed: he was still unable to hold up his head. He also showed bilateral congenital horizontally narrow eyes with drooping eyelids (blepharoptosis), heterochromia iridis, and dystopia canthorum (W-index: 2.24) (Fig. 1a). Interview and visual inspection did not find musculoskeletal abnormalities or limits of mobility in the limbs. His defecation was normal and no intestinal signs indicating constipation, disorder of peristalsis, or obstruction were apparent by interview and auscultation. Oto-acoustic emission, conditioned orientation response audiometry, and auditory brainstem response revealed severe hearing loss (Additional file 1). Computed tomography of the temporal bone revealed hypoplasia of the semicircular canals and cochlea in the bilateral ears (Additional file 2). None of the members of his family had any of the symptoms found in the proband (Additional file 3).

At 5 months of age, the proband developed an attack of generalized seizures. Initially, the seizures lasted for a few seconds and occurred once a day. In the following 2 weeks, both the frequency and duration of the seizures increased. 2 weeks after the seizures started, the proband had a systemic clonic seizure that lasted for about 4 min and the next day he had a longer clonic seizure with eye deviation to the right for about 12 min. Although obvious abnormal findings were not observed in the electroencephalogram, epilepsy was suspected. Sodium valproate was started 3 days after the 4-min-long seizure. Since then, the seizures have stopped for 3 years even after sodium valproate was stopped 2 years after the start of prescription. At the age of 12 months, he started holding up his head on his own, and, at 16 and 20 months, he started pulling up on things and walking along against a wall, respectively. Magnetic resonance imaging (MRI) performed at 8 months of age revealed delayed myelination of the frontal lobe on both T1- and T2-weighted imaging (Fig. 1b and c). However, follow-up MRI performed at 18 months of age demonstrated almost normal myelination (Fig. 1d and e).

Genomic DNA was extracted from whole blood and subjected to whole exome sequencing analysis using SureSelect Human All Exon V5 (Agilent Technologies, CA, USA). The captured DNA was subjected to the massively parallel sequencing system (HiSeq2000, Illumina, CA, USA). Paired-end read sequences were mapped and quality-checked with StrandNGS (Strand Genomics, CA, USA) using the reference sequence hg19/GRCh37. Among 88,273 variants detected in the patient and parents, 263 variants predicted to affect amino acid residues and detected in the patient with minor allele frequency (MAF) < 0.5% in public databases [6–9] were extracted and proceeded for segregation analysis within the family. Finally, eight variants of seven genes were selected as possible pathogenic mutations (Additional file 4). Of these, the *SOX10* mutation (c.652G > T, p.G218*) was absent from public databases, i.e. novel. *SOX10* has been previously reported as a responsible gene for PCWH, WS 2E (OMIM: #611584), and 4C (#613266). Since multiple nonsense mutations of *SOX10* have already been reported to cause PCWH or WS2/4 [2], the mutation detected in this case was also considered a pathogenic mutation. The heterozygous nonsense mutation in *SOX10* was in exon 3 (Additional file 5). Sanger sequencing validated the presence of the mutation in the proband, and the mutation was not present in his parents (Additional file 6).

## Discussion

WS1 can be distinguished from WS2 by the presence of dystopia canthorum [1]. About 96% of cases of WS1 are due to *PAX3* mutations [2, 3]. It has been suggested that about 3% of cases who were not associated with mutations in *PAX3* had W-index scores that were diagnostic of WS1 [10]. Indeed, although our proband was clinically classified as having WS1, whole exome sequencing did not reveal any mutation in *PAX3*; instead, a novel *SOX10* mutation was found. These findings suggest that *SOX10* mutations are one of the causes of WS1.

Truncating mutations often inactivate gene function, either because they produce truncated protein products or because there is a significant decrease in cytoplasmic mRNA abundance by nonsense-mediated mRNA decay (NMD). When a truncating mutation occurs at a nucleotide located in the last coding exon or less than 50–55 nucleotides upstream of the last coding exon, the truncated mRNA is not recognized and the NMD is escaped [11]. Then, the mutant protein is synthesized and acts as a dominant negative protein that impairs the function of the wild-type SOX10, resulting in the neurological phenotypes [12, 13]. To date, the neurological symptoms associated with *SOX10* have been reported only in the patients with truncating mutations in the last exon (exon 4 in NM_006941) of *SOX10* [9]. The present patient had a novel nonsense mutation in exon 3, 45 nucleotides upstream from the 5′ end of exon 4. Thus, this is the first case to show the neurological phenotypes due to the nonsense mutation in exon 3 of *SOX10* (Additional file 5).

Previous reports stated that the neurological symptoms of WS due to delayed myelination are congenital and irreversible [3–5]. However, the present report suggests that, while patients with a nonsense mutation in *SOX10* that is proximal to the 3′ end of exon 3 can also exhibit delayed myelination, they may develop severe attacks of generalized seizures that can be improved because the immature myelination can recover later. Long-term follow-up is required for these patients because recurrence of seizure attacks may occur in the future. These findings suggest that they may be clinical characteristics of WS that arise from a nonsense mutation in exon 3 of *SOX10*. It is also possible to speculate that modifier genes that complement or repair immature myelination played a role. In conclusion, when patients of WS show mild prodromal neurological symptoms, such as delayed motor development and/or blepharoptosis, the clinician should be aware of the possibility that severe attacks of generalized seizures may follow, which may be associated with the truncating mutation in exon 3 of *SOX10*.

## Additional files

Additional file 1: Hearing test results of the proband. OAE (a) and COR (b) audiometry revealed severe hearing loss. On the ABR test, neither the right (c) nor the left (d) ear responded to click sound stimulation at 105 dBnHL. Lt, left; Rt, right. (DOCX 295 kb)

Additional file 2: Axial CT of the temporal bone. These axial CT images are the series of slices taken from the cranial side toward the caudal side (a–h: left ear; i–p: right ear). The CT imaging revealed hypoplasia of the semicircular canals and cochlea. (DOCX 1699 kb)

Additional file 3: Pedigree of the family in this study. Round and square symbols indicate females and males, respectively. The individuals who were examined and whose blood samples were collected for DNA analysis are indicated by a horizontal bar above the symbol. None of the family members other than the proband had any Waardenburg syndrome-related symptoms. P: proband. (DOCX 43 kb)

Additional file 4: Summary of the eight candidate variants in the proband that were detected by whole exome sequencing. The *SOX10* mutation was indicated in red. (DOCX 20 kb)

Additional file 5: Schematic depiction indicating the position of the detected mutation in *SOX10*. The translated regions are indicated by black filled rectangles in the upper line. (DOCX 176 kb)

Additional file 6: Electropherograms showing partial sequences of *SOX10*. Subject II:3 (a) has a homozygous G (indicated by an arrow) in the first nucleotide of codon 218, which encodes glycine (G). The proband (b) has a heterozygous G to T transition (arrow) at the same position that causes the glycine (G) at codon 218 to be replaced with a stop codon (*). This causes premature termination of protein synthesis. (DOCX 179 kb)

## Abbreviations

MRI: Magnetic resonance imaging
NMD: nonsense-mediated mRNA decay
PCWH: Peripheral demyelinating neuropathy, Central dysmyelinating leukodystrophy, Waardenburg syndrome, Hirschsprung disease
WS: Waardenburg syndrome

## References

[1] Read AP, Newton VE (1997). Waardenburg syndrome. J Med Genet.

[2] Inoue K, Khajavi M, Ohyama T, Hirabayashi S, Wilson J, Reggin JD (2004). Molecular mechanism for distinct neurological phenotypes conveyed by allelic truncating mutations. Nat Genet.

[3] Bondurand N, Dastot-Le F, Stanchina L, Collot N, Baral V, Marlin S (2007). Deletions at the SOX10 gene locus cause Waardenburg syndrome types 2 and 4. Am J Hum Genet.

[4] Chaoui A, Watanabe Y, Touraine R, Baral V, Goossens M, Pingault V (2011). Identification and functional analysis of SOX10 missense mutation in different subtypes of Waardenburg syndrome. Hum Mutat.

[5] Sznajer Y, Coldéa C, Meire F, Delpierre I, Sekhara T, Touraine RL (2008). A de novo SOX10 mutation causing severe type 4 Waardenburg syndrome without Hirschsprung disease. Am J Med Genet A.

[6] Database of Single Nucleotide Polymorphisms (dbSNP). http://www.ncbi.nlm.nih.gov/snp/. Accessed 16 Dec 2016.

[7] 1000Genomes. http://www.1000genomes.org. Accessed 16 Dec 2016.

[8] NHLBI Exome Variant Server (ESP6500). http://evs.gs.washington.edu/EVS/. Accessed 16 Dec 2016.

[9] Human Genetic Variation Database (HGVD). http://www.genome.med.kyoto-u.ac.jp/SnpDB/index.html. Accessed 16 Dec 2016.

[10] Farrer LA, Arnos KS, Asher JH Jr, Baldwin CT, Diehl SR, Friedman TB, et al. Locus Heterogeneity for Waardenburg Syndrome Is Predictive of Clinical Subtypes. Am J Hum Genet 1994;55:728–737. PMC1918288 7942851

[11] Kervestin S, Jacobson ANMD (2012). A multifaceted response to premature translational termination. Nat Rev Mol Cell Biol.

[12] Pingault V, Girard M, Bondurand N, Dorkins H, Van Maldergem L, Mowat D (2002). SOX10 mutations in chronic intestinal pseudo-obstruction suggest a complex physiopathological mechanism. Hum Genet.

[13] Inoue K, Shilo K, Boerkoel CF, Crowe C, Sawady J, Lupski JR (2002). Congenital hypomyelinating nuropathy, central dysmyelination, and Waardenburg-Hirschsprung disease: phenotypes linked by SOX10 mutation. Ann Neurol.

