# Supplementary figures and images for: A case report of reversible generalized seizures in a patient with Waardenburg syndrome associated with a novel nonsense mutation in the penultimate exon of SOX10
Source: BMC Pediatr. 2018 May 23;18:171. doi: 10.1186/s12887-018-1139-2 (PMC5966879; doi:10.1186/s12887-018-1139-2)

Additional file 1

**a**
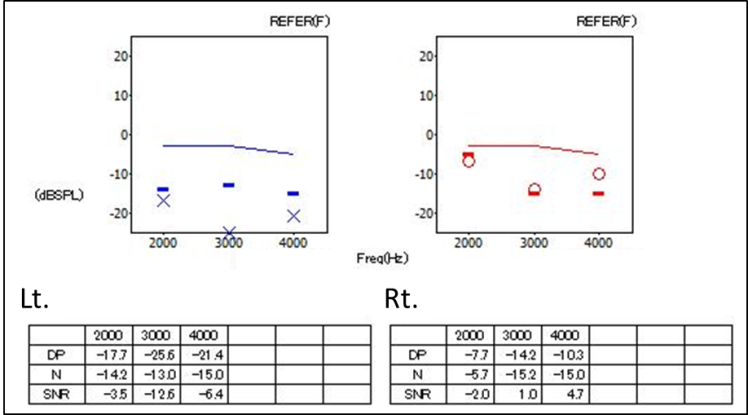


**b**
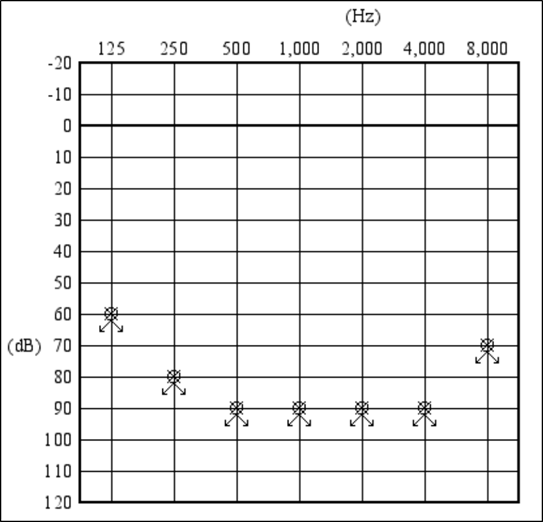


**c**
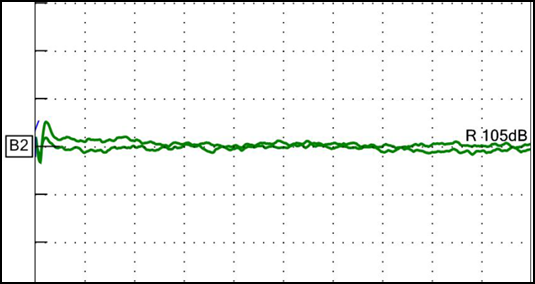


**d**
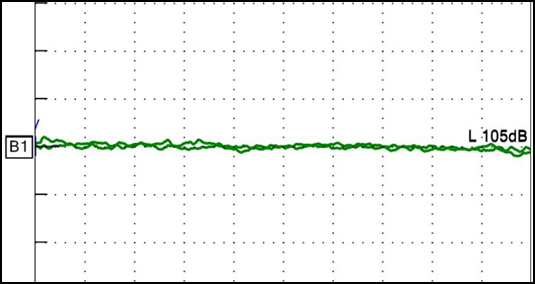

Supplement: Supplementary file 1 — Hearing test results of the proband. OAE (a) and COR (b) audiometry revealed severe hearing loss. On the ABR test, neither the right (c) nor the left (d) ear responded to click sound stimulation at 105 dBnHL. Lt, left; Rt, right. (DOCX 295 kb) [file 12887_2018_1139_MOESM1_ESM.docx]

Additional file 2

**a**
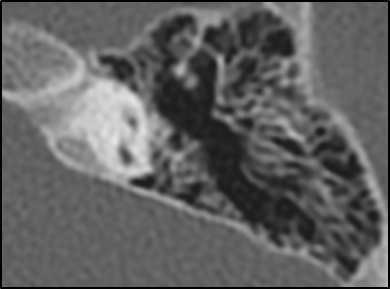
 **b**
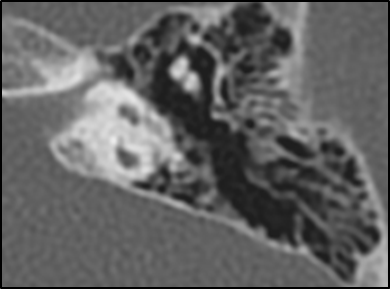
 **c**
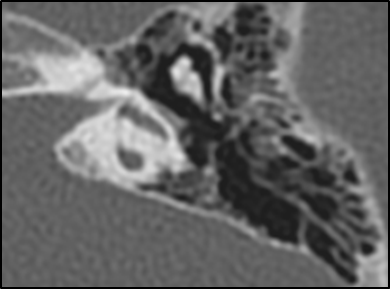


**d**
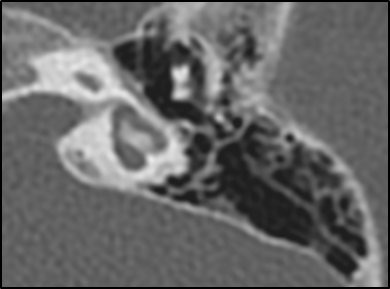
 **e**
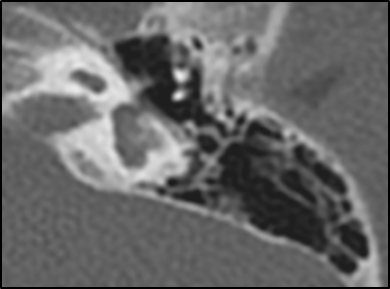
 **f**
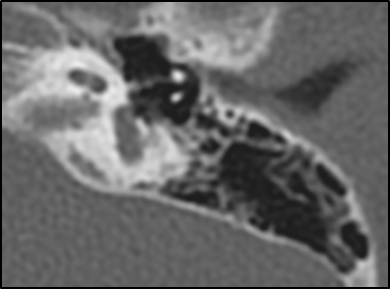


**g**
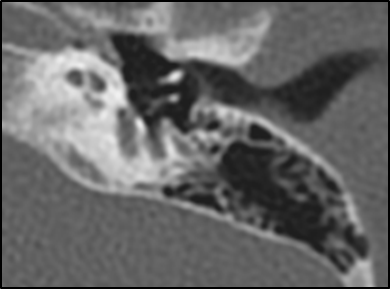
 **h**
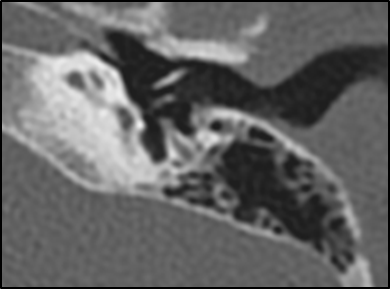


**i**
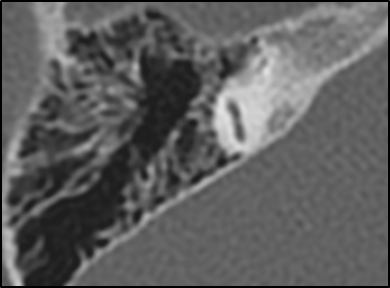
 **j**
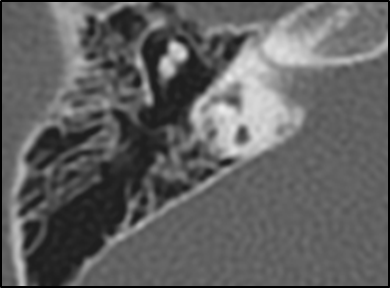
 **k**
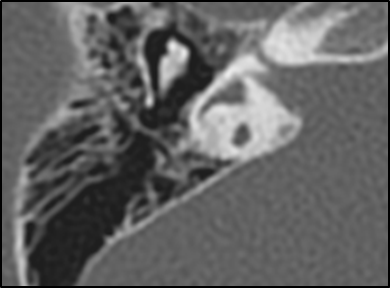


**l**
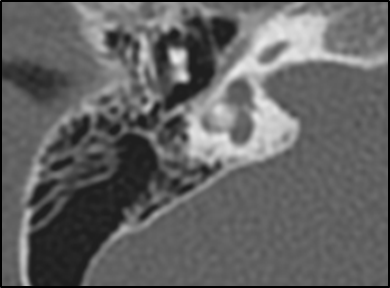
 **m**
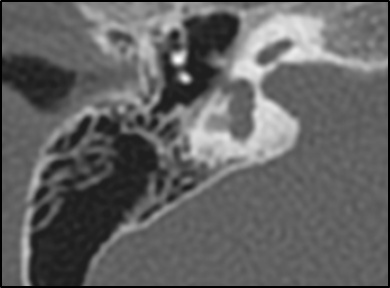
 **n**
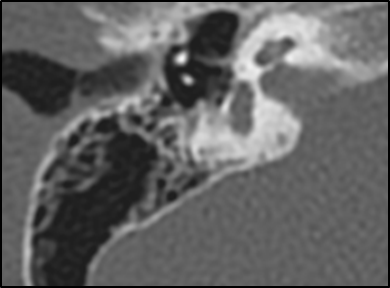


**o**
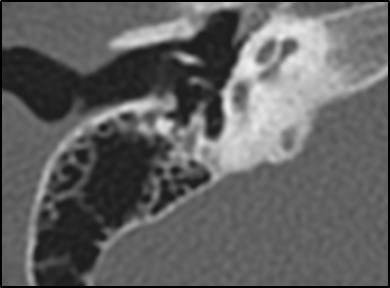
 **p**
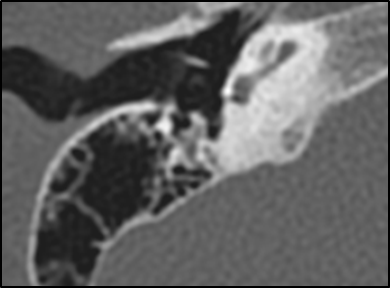

Supplement: Supplementary file 2 — Axial CT of the temporal bone. These axial CT images are the series of slices taken from the cranial side toward the caudal side (a–h: left ear; i–p: right ear). The CT imaging revealed hypoplasia of the semicircular canals and cochlea. (DOCX 1699 kb) [file 12887_2018_1139_MOESM2_ESM.docx]

Additional file 3


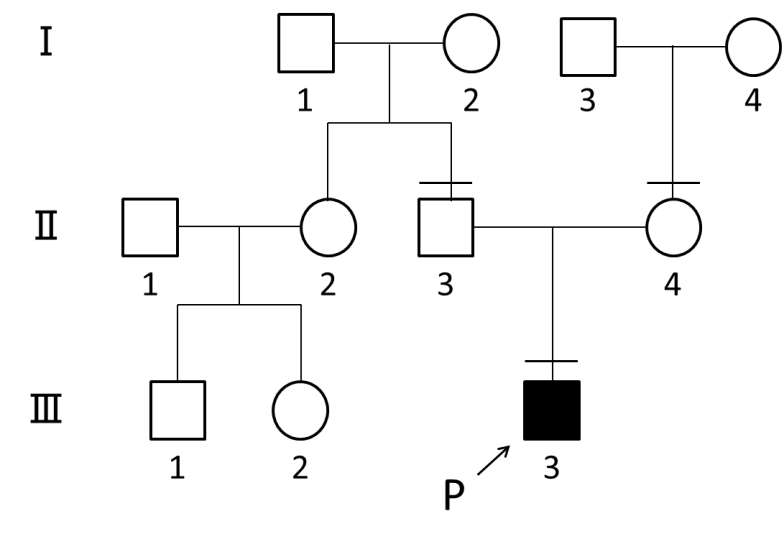

Supplement: Supplementary file 3 — Pedigree of the family in this study. Round and square symbols indicate females and males, respectively. The individuals who were examined and whose blood samples were collected for DNA analysis are indicated by a horizontal bar above the symbol. None of the family members other than the proband had any Waardenburg syndrome-related symptoms. P: proband. (DOCX 43 kb) [file 12887_2018_1139_MOESM3_ESM.docx]

Additional file 6

**a**
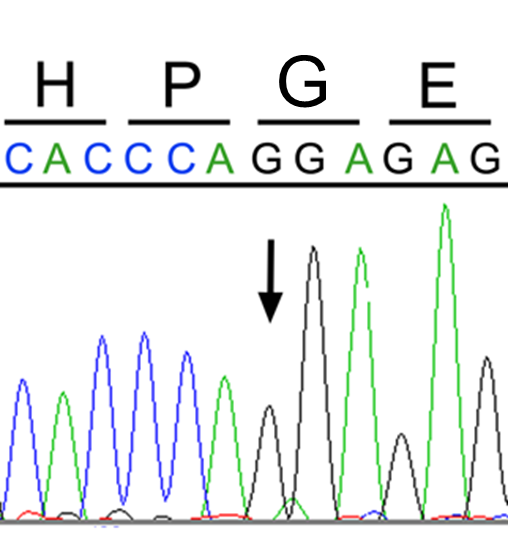
 **b**
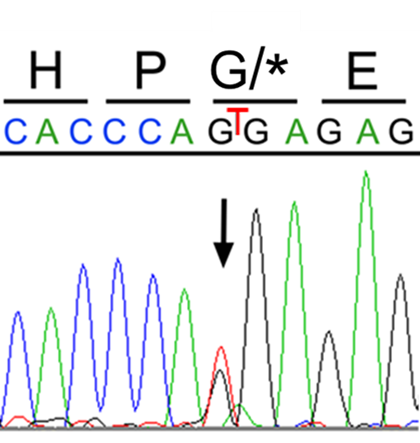

Supplement: Supplementary file 6 — Electropherograms showing partial sequences of SOX10. Subject II:3 (a) has a homozygous G (indicated by an arrow) in the first nucleotide of codon 218, which encodes glycine (G). The proband (b) has a heterozygous G to T transition (arrow) at the same position that causes the glycine (G) at codon 218 to be replaced with a stop codon (*). This causes premature termination of protein synthesis. (DOCX 179 kb) [file 12887_2018_1139_MOESM6_ESM.docx]
