# Supplementary material for: A case report of reversible generalized seizures in a patient with Waardenburg syndrome associated with a novel nonsense mutation in the penultimate exon of SOX10
Source: BMC Pediatr. 2018 May 23;18:171. doi: 10.1186/s12887-018-1139-2 (PMC5966879; doi:10.1186/s12887-018-1139-2)
Supplement: Supplementary file 4 — Summary of the eight candidate variants in the proband that were detected by whole exome sequencing. The SOX10 mutation was indicated in red. (DOCX 20 kb) [file 12887_2018_1139_MOESM4_ESM.docx]

Additional file 4

Summary of the eight candidate variants in the proband that were detected by whole exome sequencing.

| chr | Gene | Position in cDNA | Amino Acid Change | inheritence | dbSNP Id | MAF in HGVD | MAF in 1000G | MAF in ESP6500 |
| --- | --- | --- | --- | --- | --- | --- | --- | --- |
| 4 | *RNF212* | c.725A>T | p.Q242L | *de novo* | rs146994272 | n/a | n/a | n/a |
| 10 | *DNMBP* | c.3814C>T | p.Q1272* | *de novo* | n/a | n/a | n/a | n/a |
| 12 | *TRPV4* | c.1584+3insT | - | *de novo* | n/a | n/a | n/a | n/a |
| 15 | *GJD2* | c.244C>T | p.Q82* | *de novo* | n/a | n/a | n/a | n/a |
| 16 | *SRRM2* | c.4364C>T | p.S1455L | *de novo* | n/a | n/a | n/a | 1.42E-04 |
| 17 | *TNFRSF13B* | [c.92delT];  [c.740C>T] | [p.M31Rfs];  [p.A181T] | compound hetero | rs150068036,rs374314027/rs149635611 | n/a, 0.004 | n/a, 0.0005 | n/a |
| **22** | ***SOX10*** | **c.652G>T** | **p.G218*** | ***de novo*** | **n/a** | **n/a** | **n/a** | **n/a** |

Abbreviations: chr, chromosome; dbSNP, the single nucleotide polymorphism database; MAF, minor allele frequency; HGVD, human genetic variation database; 1000G, 1000 genomes database; ESP6500, NHLBI Exome Sequencing Project; n/a, not available.
