# Supplementary material for: A case report of reversible generalized seizures in a patient with Waardenburg syndrome associated with a novel nonsense mutation in the penultimate exon of SOX10
Source: BMC Pediatr. 2018 May 23;18:171. doi: 10.1186/s12887-018-1139-2 (PMC5966879; doi:10.1186/s12887-018-1139-2)
Supplement: Supplementary file 5 — Schematic depiction indicating the position of the detected mutation in SOX10. The translated regions are indicated by black filled rectangles in the upper line. (DOCX 176 kb) [file 12887_2018_1139_MOESM5_ESM.docx]

Additional file 5


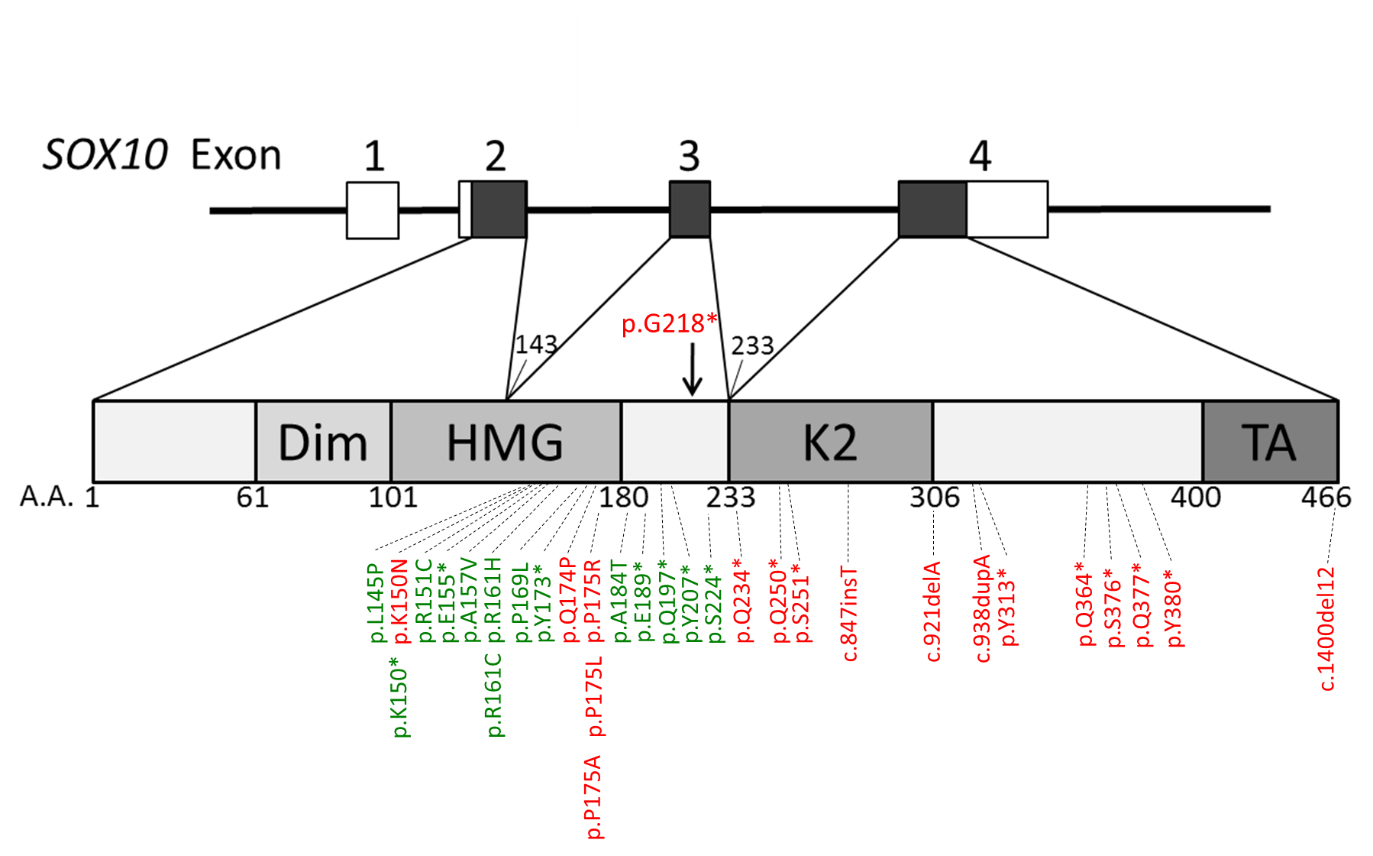


Introduction: The location of the p.G218* mutation is indicated by the solid arrow. The number of amino acids at the junction between exons is shown in the lower line. Positions of the previously reported mutations on the exon 3 or with the neurological symptoms associated with SOX10 are also indicated. Green indicates a WS phenotype, red indicates a PCWH phenotype. Abbreviations: Dim, DNA-dependent dimerization domain; HMG, high-mobility-group domain; K2, context-dependent transactivation domain; TA, main transactivation domain. References (in main text or PubMed ID) for each mutation are: p.L145P, K150N, R161H, P175A, P175L, P175R [4]; p.K150*, E155* (27938609); p.R151C (23643381); p.A157V (18348274); p.R161C (25077900); p.P169L (25256313); p.Y173*, c.921delA (20127975); p.Q174P (19208381); p.A184T (26077850); p.E189* (9462749); p.Q197* (27759048); p.Y207* (10077527); p.S224* (22246888); p.Q234* [12]; p.Q250* [13]; p.S251*, Y313* (10762540); c.847insT, c.938dupA, p.Q364* [2]; p.S376* (25991456); p.Y380* (28534044); c.1400del12 (10482261).
